# *Angelica gigas* NAKAI and Its Active Compound, Decursin, Inhibit Cellular Injury as an Antioxidant by the Regulation of AMP-Activated Protein Kinase and YAP Signaling

**DOI:** 10.3390/molecules27061858

**Published:** 2022-03-13

**Authors:** Yu-Rim Song, Boyun Jang, Sung-Min Lee, Su-Jin Bae, Seon-Been Bak, Young-Woo Kim

**Affiliations:** School of Korean Medicine, Dongguk University, Gyeongju 38066, Korea; khf4856@naver.com (Y.-R.S.); bboyunjang@naver.com (B.J.); leesungmin79@gmail.com (S.-M.L.); realsujin@naver.com (S.-J.B.); skyblue014@gmail.com (S.-B.B.)

**Keywords:** AMPK, YAP, *Angelica gigas* NAKAI, neuropsychiatry, mitochondria, dementia

## Abstract

Natural products and medicinal herbs have been used to treat various human diseases by regulating cellular functions and metabolic pathways. *Angelica gigas* NAKAI (AG) helps regulate pathological processes in some medical fields, including gastroenterology, gynecology, and neuropsychiatry. Although some papers have reported its diverse indications, the effects of AG against arachidonic acid (AA)+ iron and carbon tetrachloride (CCl_4_) have not been reported. In HepG2 cells, AA+ iron induced cellular apoptosis and mitochondrial damage, as assessed by mitochondrial membrane permeability (MMP) and the expression of apoptosis-related proteins. On the other hand, AG markedly inhibited these detrimental phenomena and reactive oxygen species (ROS) production induced by AA+ iron. AG activated the liver kinase B1 (LKB1)-dependent AMP-activated protein kinase (AMPK), which affected oxidative stress in the cells. Moreover, AG also regulated the expression of yes-associated protein (YAP) signaling as mediated by the AMPK pathways. In mice, an oral treatment of AG protected against liver toxicity induced by CCl_4_, as indicated by the plasma and histochemical parameters. Among the compounds in AG, decursin had antioxidant activity and affected the AMPK pathway. In conclusion, AG has antioxidant effects in vivo and in vitro, indicating that natural products such as AG could be potential candidate for the nutraceuticals to treat various disorders by regulating mitochondrial dysfunction and cellular metabolic pathways.

## 1. Introduction

Oxidative stress plays a pivotal role in several chronic diseases such as cancer, diabetes, fatty liver disease, aging, arthritis and neurological disease. The dysfunction of lipid metabolism affects cell membranes and causes the accumulation of lipids in the blood, which is linked to oxidative stress and inflammatory responses. Many studies reported that oxidative stress is related to inflammation and carcinogenesis and is particularly linked to DNA damage [1,2]. Studies have reported chronic inflammation and oxidative stress in liver diseases [3,4,5,6,7].

Reactive oxygen species (ROS) are byproducts, resulting in normal cellular metabolism, and can have beneficial effects against pathogens and metabolic disorders [8,9,10]. ROS released in limited amounts under controlled conditions help prevent cell death by acting as cellular signaling mediators. ROS play decisive roles in eliminating unnecessary cellular components, impaired organelles, and pathogens. ROS are normally generated to remove the broken debris of cellular organelles and fix the disorders of cells and pathogens [11].

The liver is the main organ for lipid metabolism and iron storage. Cotreatment of arachidonic acid (AA)+ iron synergistically generates excessive ROS production through mitochondrial membrane damage [12]. The mitochondrial membrane is highly susceptible to oxidative damage by ROS. The mitochondrion is the pivotal organelle that produces ATP, which is used as essential energy in cells. Oxidative stress interferes with mitochondrial functions through an impairment of the membrane [13].

AMP-activated protein kinase (AMPK) is an energy sensor at the cellular level and acts as a key regulator of energy metabolism [12]. Yes-associated protein (YAP) is an energy pathway that regulates the homeostasis of energy levels. In cellular energy stress, the YAP serine residue is phosphorylated and regulated directly by AMPK activation and partially controlled by indirect AMPK-dependent large tumor suppressor (LATS) activation. Therefore, YAP activity is inhibited [14]. A previous study reported that AMPK-mediated YAP inhibition could suppress carcinogenic transformation in cells by using LATS-null cells with high YAP activity. YAP inhibition by AMPK in energy-limited situations does not allow cell growth because it consumes energy in the proliferation process, which may contribute to tumor suppression [14].

*Angelica gigas* NAKAI (AG) is used traditionally in East Asian countries, including Korea. It is a medicinal herb used to treat poor circulation, articular rheumatism, anemia, infection, inflammation, the common cold, abdominal pain, migraine and menstrual disorders, amenorrhea, dysmenorrhea, and premenstrual syndrome [15,16]. AG has been used to treat hepatic steatosis, hyperlipidemia, and hypercholesterolemia [17]. It contains many coumarin compounds, such as decursin and nodakenin [18]. 

This study investigated the cell-protective effect of AG in vitro and in vivo against AA+ iron and a carbon tetrachloride (CCl_4_) treatment, respectively. The underlying antioxidant mechanism of AG was observed. AG phosphorylated AMPK and its downstream cellular signals through liver kinase B1 (LKB1) activation. In addition, the activated AMPK induced the inhibition of YAP, suppressing the movement of YAP to nuclei partially through activated LATS. These experiments show that the anti-oxidant effect of AG acts through AMPK-mediated YAP/LATS signaling pathways.

## 2. Materials and Methods

### 2.1. Chemicals and Reagents

Ferric nitrate (Fe, iron), rhodamine 123 (Rh 123), 3-(4,5-dimethylthiazol-2-yl)-2,5-diphenyl-tetrazolium bromide (MTT), 2’,7’-dichlorofluorescein diacetate (DCFH-DA), Harri’s hematoxylin and eosin (H&E) were purchased from Sigma (St. Louis, MO, USA). AA was obtained from Calbiochem (San Diego, CA, USA). Anti-caspase3, anti-B-cell lymphoma-extra large (Bcl-xL), anti-β-actin, anti-phospho-ACC, anti-phospho-AMPKα, anti-phospho-liver kinase B1 (LKB1), anti-phospho-LATS1, anti-YAP, and anti-phospho-YAP antibodies were supplied by Cell Signaling Technology (Danvers, MA, USA). AG was extracted in the boiled water using the medicinal herb of *Angelica gigas* NAKAI, which is produced by the pharmaceutical company Daewon pharmacy (Daegu, Korea) [19].

### 2.2. Cell Culture

HepG2, Hep3B, and HeLa cells were obtained from American Type Culture Collection (ATCC, Rockville, MD, USA). The cells were maintained in Dulbecco’s modified Eagle’s medium liquid (DMEM) with high glucose levels, 10% fetal bovine serum (FBS), 50 units/mL penicillin, and 50 μg/mL streptomycin at 37 °C in a humidified atmosphere containing 5% CO_2_. For all experiments, the cells were starved for 12 h in FBS-free DMEM with high glucose levels.

### 2.3. MTT Assay

HepG2 cells were plated in 48-well culture plates and incubated in an FBS-free medium for 12 h. The cells were incubated with 10, 30, 100, and 300 μg/mL AG for 1 h, followed by a treatment with AA (10 μM) for 12 h and then iron (5 μM) for 6 h. Cell viability was defined as relative to the untreated control (i.e., viability (% of control) = 100 × (absorbance of the treated sample)/(absorbance of control)) as previously described [19,20].

### 2.4. Immunoblot Analysis

The cells treated as described above were lysed using an RIPA buffer at 4 °C, and the supernatant was collected by centrifugation at 15,000 rpm at 4 °C for 30 min. A BCA protein assay kit (Thermo Fisher Scientific Inc., Waltham, MA, USA) was used to quantify proteins in cell lysates, as previously described [20]. The bands were developed by using an ECL reagent and a Chemidoc image analyzer (Vilber Lourmat, Collégien, France).

### 2.5. Reactive Oxygen Species (ROS) Production Measurement

HepG2 cells were plated in 96-well black plates at a density of 1 × 10⁴ per well, as previously described [21]. Subsequently, they were treated as described above and incubated with 10 μM DCFH-DA for 30min at 37 °C. 

### 2.6. Mitochondrial Membrane Potential (MMP) Measurement

MMP was measured by flow cell analysis by staining with Rh 123 as previously described [22]. The HepG2 cells were treated as described and collected by staining with 0.05μg/mL Rh 123 for 1 h at 37 °C. Fluorescence was detected by BD Accuri C6 Plus Flow Cytometer (BD Biosciences, Franklin Lakes, NJ, USA).

### 2.7. Animals and Treatment

Male C57BL/6N mice (6 weeks old, 20–21 g) were purchased from Charles River Orient Bio (Seongnam, Korea). Mice were divided randomly into five groups: vehicle-treated control, CCl_4_, CCl_4_ + AG 100 mg/kg, and CCl_4_ + AG 300 mg/kg. The mice were orally administered with either AG (100, 300 mg/kg, dissolved in water) or the vehicle (only water) once daily for three days. A single intraperitoneal injection of 0.5 mL/kg of CCl_4_ mixed with olive oil (1:1, *v*/*v*) was administered 2 h after the last dose of AG. Twenty-four hours after administration, the mice were sacrificed, and the livers were excised. Blood was collected, and the serum was separated by centrifugation (3000 rpm, 15 min) before an analysis of alanine aminotransferases (ALT) as previously described [22]. Formalin-fixed liver tissue was made into a paraffin block using tissue processing and embedding procedures to stain H&E as previously described [22]. 

### 2.8. Statistical Analysis

The data obtained from independent experiments were analyzed by using a one-way analysis of variance (ANOVA) or a two-tailed Student’s t-test. The criterion for statistical significance was set to *p* < 0.05, *p* < 0.01, or *p* < 0.001

## 3. Results

### 3.1. Effects of AG on AA+iron-Induced Oxidative Stress

**Figure 1:**
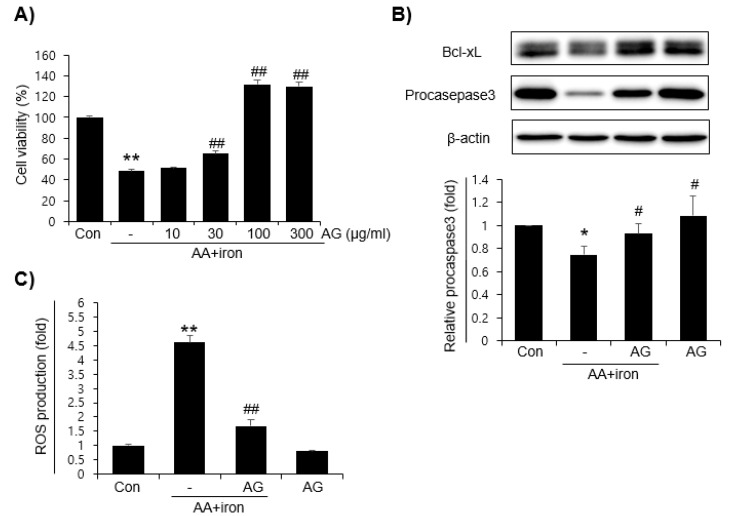
Effect of AG on arachidonic acid (AA)+iron-induced cytotoxicity in HepG2 cells (**A**) Cell viability was assessed by the MTT assay. HepG2 cells were pretreated with AG (10, 30, 100, and 300 μg/mL) for 1 h. Subsequently, the cells were treated with AA (10 μM) for 12 h and iron (5 μM) for 6 h. (**B**) Immunoblot analysis of apoptosis-associated proteins, Bcl-xL and procaspase3. HepG2 cells were treated with AG (100 μg/mL) for 1 h, AA (10 μM) for 12 h, and iron (5 μM) for 1 h. (**C**) ROS production measurements. HepG2 cells were stained for 30min on DCFH-DA (10 μM) after incubation for 1 h AG (100 μg/mL), 12 h AA (10 μM) and 1 h iron (5 μM). All data represent means ± SD of three independent experiments (* *p* < 0.05 and ** *p* < 0.01 vs. control group; # *p* < 0.05 and ## *p* < 0.01 vs. AA+iron-treated group). AA, arachidonic acid; AG, *Angelica gigas* NAKAI.

The toxicity of AG according to the concentrations (10, 30, 100, and 300 μg/mL) was tested on HepG2 cells by using a MTT assay. AG did not affect cell viability, as measured by the MTT assay (data not shown). Next, the effect of AG was investigated by inducing oxidative stress caused by AA+iron in HepG2 cells by an MTT assay. HepG2 cells were treated with increasing concentrations (10, 30, 100, and 300 μg/mL) of AG, and cell viability was measured by using an MTT assay. Treatment with AA+iron reduced MTT levels significantly, whereas a treatment with AG protected the effect of AA+iron in a concentration-dependent manner (Figure 1A). As the maximal cell viability of AG was shown at 100 μg/mL, this concentration was used for further experiments. Next, the levels of apoptosis-related protein markers, procaspase-3 and Bcl-xL, were measured by immunoblot analysis to further investigate the protective effect of FLL against AA+iron-induced cytotoxicity further. Treatment of AA+iron reduced the levels of procaspase-3 and Bcl-xL markedly. This effect was blocked by the AG pretreatment (Figure 1B). The effects of AG on the oxidative stress produced by AA+iron were investigated. There was no increase in ROS production in the cells treated with AG alone, and treatment with AA+iron increased ROS levels significantly. Pretreatment of AG strongly reduced ROS generation by AA+iron (Figure 1C).

### 3.2. Effect of AG on Mitochondrial Damage 

**Figure 2:**
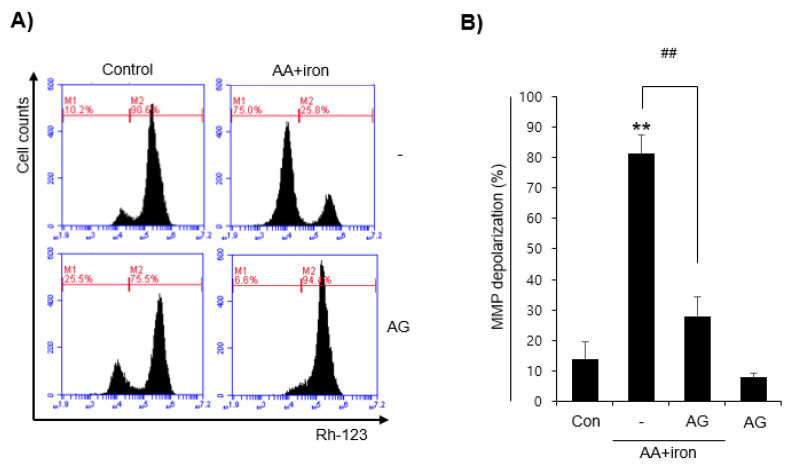
Effects of AG on the mitochondrial membrane potential (MMP) (**A**) Mitochondria protective effect of AG by flow cell analysis. HepG2 cells were treated as described in Fig. 1. The cells were then incubated with Rh 123 (0.05 μg/mL) for 1 h. (**B**) Relative proportion of low Rh 123 intensity was expressed as the mean ± SD of three independent experiments (** *p* < 0.01 vs. control group; ## *p* < 0.01 vs. AA+iron-treated group). AA, arachidonic acid; AG, *Angelica gigas* NAKAI.

The mitochondria are the major source of intracellular ROS and oxidative stress in all organs of the human body. This study investigated mitochondrial dysfunction relative to AA+iron by flow cytometry by using Rh123, a lipophilic cationic dye capable of detecting the changes in MMP. Compared to the control group, the AA+iron-treated group shifted to the left, increasing the RN1 fraction, which comprises rhodamine 123 negative cells. AG treated group showed no significant change compared to the control group. The AG treatment prevented an increase in the RN1 fraction induced by AA+iron (Figure 2A,B). These results suggest that AG protects against AA+iron-induced mitochondrial dysfunction and MMP loss.

### 3.3. Effect of AG on LKB1-AMPK Pathway Activation

**Figure 3:**
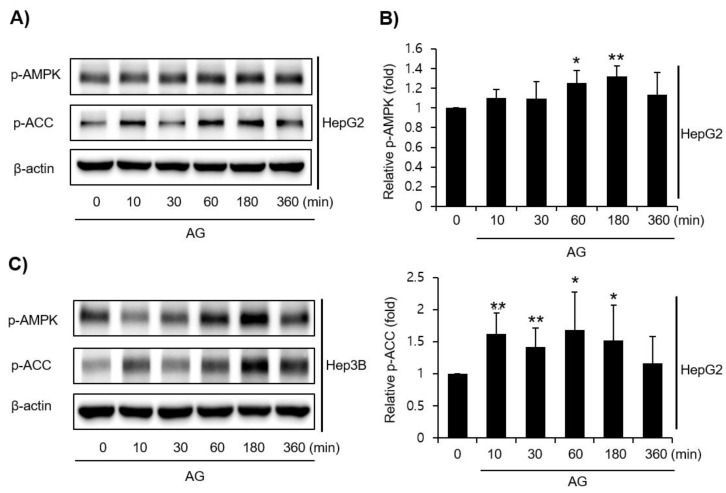
Effect of AG on AMPKα activation. Western blotting of AMPK-signaling pathway molecules in HepG2 and Hep3B cells. (**A**) HepG2 cells were incubated with AG (100 μg/mL) for the indicated periods. β-actin was used as a loading control. (**B**) Relative protein levels in HepG2. The data represent the means ± SD of three independent experiments (* *p* < 0.05 and ** *p* < 0.01 vs. control group). (**C**) Hep3B cells were incubated with AG (100 μg/mL) for indicated periods. AA, arachidonic acid; AG, *Angelica gigas* NAKAI.

**Figure 4:**
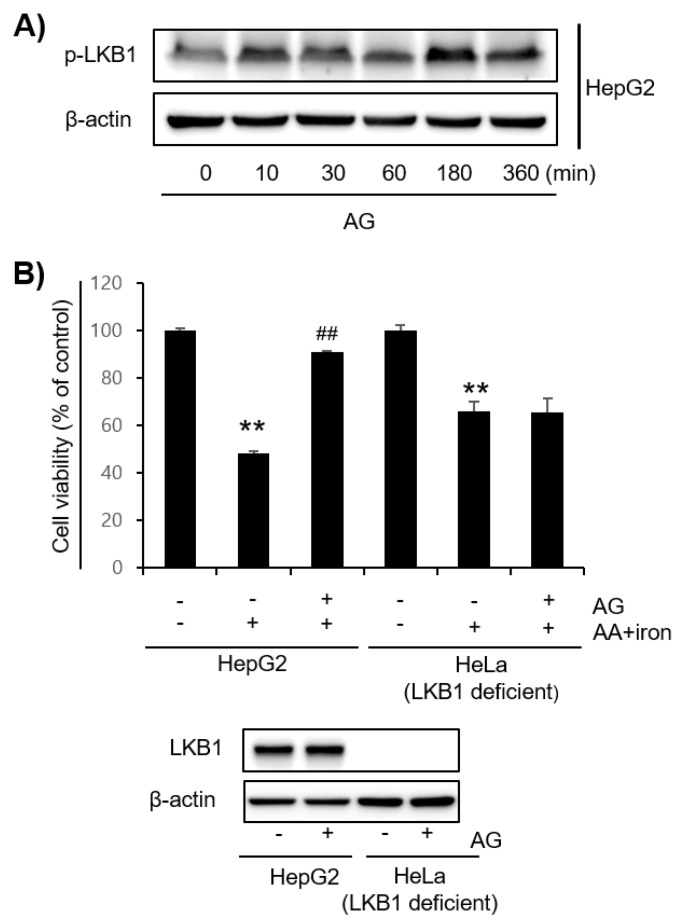
Expression effect of LKB1 by AG (**A**) Western blotting of phosphorylation of LKB1, an upstream regulator of the AMPK signaling pathway. HepG2 cells were incubated with AG (100 μg/mL) for the indicated times. (**B**) Effect of AG on AA + iron-induced apoptosis on HepG2 and LKB1-deficient HeLa cells. The cells were pretreated with AG (100 μg/mL) for 1 h, followed by AA (10 μM) for 12 h and iron (5 μM) for 6 h. The MTT assay was performed for cell viability. LKB1-deficient was confirmed by Western blotting. The cells were treated with AG (100 μg/mL) for 1 h. The graph represents the means ± SD of three independent experiments (** *p* < 0.01 vs. control group; ## *p* < 0.01 vs. AA + iron-treated group). AA, arachidonic acid; AG, *Angelica gigas* NAKAI.

AMPK activation was measured by Western blotting to investigate the mechanisms responsible for the effects of AG. In HepG2 and Hep3B cells, the AG treatment induced the phosphorylation of AMPK, which peaked at 1–3 h. In addition, these two cells also showed that ACC, a major downstream target of AMPK, was phosphorylated upon treatment with AG (Figure 3A–C). LKB1, an upstream kinase of AMPK, was also phosphorylated in HepG2 cells by the AG treatment (Figure 4A). The MTT assay showed that AG does not appear to protect LKB1-deficient Hela cells against AA+iron-induced apoptosis. A treatment of LKB1-deficient Hela cells with AG clearly blocked LKB1, as was detected clearly in HepG2 cells (Figure 4B).

### 3.4. Effect of AG on YAP Signaling

**Figure 5:**
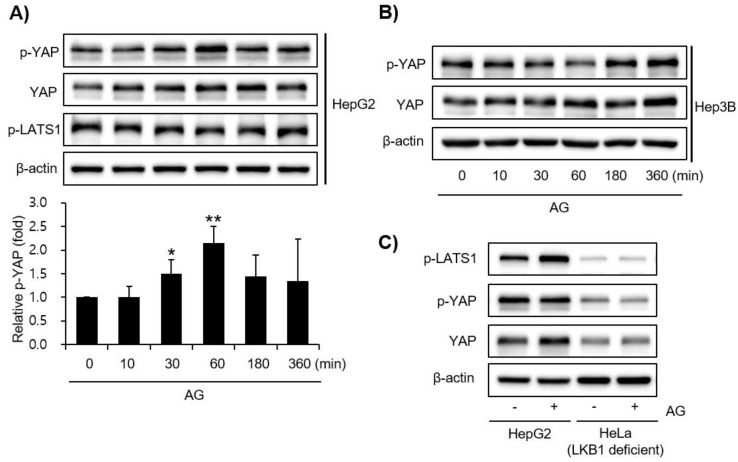
Effect of AG on YAP signal. Western blotting of YAP-signaling pathway molecules in HepG2, Hep3B, and LKB1-deficient HeLa cells. (**A**) HepG2 and (**B**) Hep3B cells were incubated with AG (100 μg/mL) for indicated times. β-actin was used as the loading control. The data represent the means ± SD of three independent experiments (* *p* < 0.05 and ** *p* < 0.01 vs. control group). (**C**) HepG2 and LKB1-deficient HeLa cells were incubated for 1 h with AG (100 μg/mL) treatment. AA, arachidonic acid; AG, *Angelica gigas* NAKAI.

The mechanisms responsible for the effects of AG were examined further by measuring YAP signaling pathway activation by Western blotting. AG increased the YAP level most when the HepG2 cells were also treated with phospho-YAP for 1 h. The Hep3B cells also showed an increase in YAP levels (Figure 5A,B). In addition, the levels of p-LATs1, p-YAP, and YAP were detected in HepG2 cells treated with AG, but the expression levels were weak in LKB1-deficient Hela cells (Figure 5C).

### 3.5. Effect of Decursin on Oxidative Stress as an Active Component in AG

**Figure 6:**
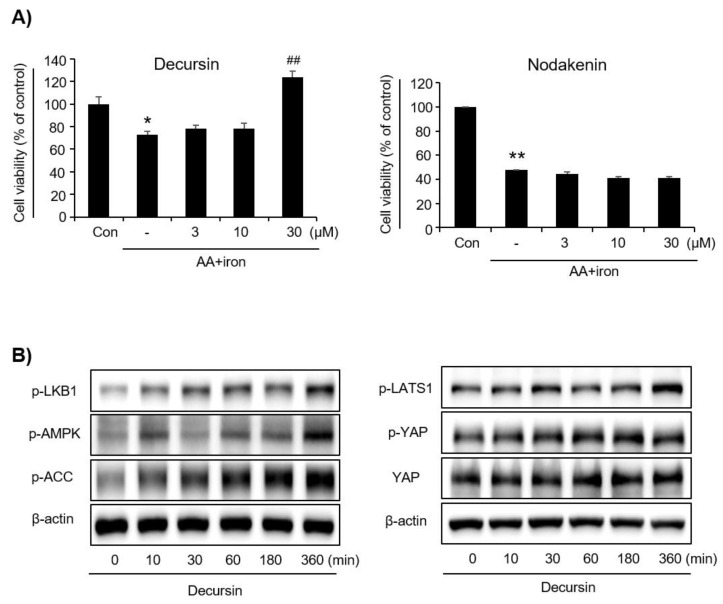
Effects of Decursin, one of the representative components. (**A**) Decursin effectively protected AA + iron-induced cell death, but Nodakenin did not. HepG2 cells were treated with 3 μM, 10 μM, and 30 μM of decursin and nodakenin for 1 h and then continuously incubated with 10 mM AA for 12 h, followed by exposure to 5 mM iron for 6 h. Cell viability was assessed using the MTT assay. Data represent the means ± SD of three independent experiments (* *p* < 0.05 and ** *p* < 0.01 vs. control group; ## *p* < 0.01 vs. AA+iron-treated group). (**B**) Effects of decursin on the AMPK activity and YAP signaling by Western blotting. The HepG2 cells were incubated with decursin (30 μM) for the indicated times. AA, arachidonic acid; AG, *Angelica gigas* NAKAI.

Decursin and nodakenin, which are representative ingredients of AG, were selected [23]. The decursin 30 μM treatment reduced AA+iron-induced apoptosis significantly, whereas nodakenin had no effect (Figure 6A). Next, decursin increased levels of LKB1, AMPK, and ACC phosphorylation in the AMPK pathway and increased the levels of LATS1 and YAP phosphorylation in YAP signaling (Figure 6B).

### 3.6. Effect of AG on CCl₄-Induced Liver Injury 

**Figure 7:**
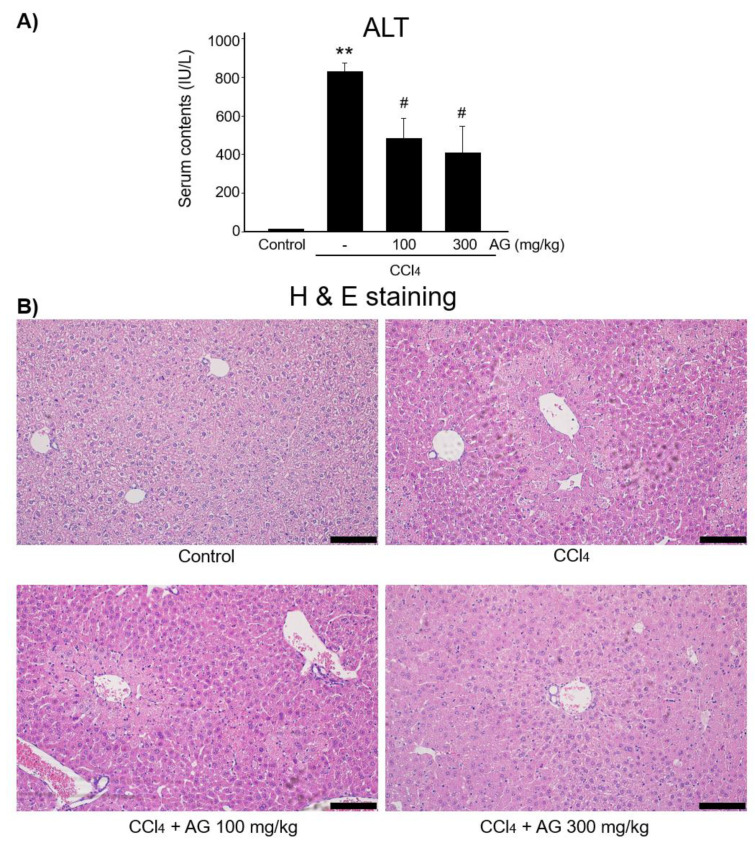
Effect of AG in CCl₄-induced liver toxicity model. (**A**) Serum ALT level. (**B**) H&E staining. Mice were treated orally with 100 mg/kg or 300 mg/kg of AG. (** *p* < 0.01 vs. control group; # *p* < 0.05 vs. CCl_4_-treated group) Scale bar = 100 μm, AG, *Angelica gigas* NAKAI.

We evaluated serum ALT levels as an indicator of liver damage induced by CCl_4_ treatment. The level of ALT in serum was significantly increased by CCl_4_ treatment. However, this pathological parameter was inhibited by pretreatment with AG at doses of 100 mg/kg and 300 mg/kg (Figure 7A). To investigate hepatoprotective effects of AG, we additionally conducted H&E staining and observed histological changes. Severe damages to liver were detected in the CCl_4_ treatment group compared to those in the vehicle-treated control group. Both 100 mg/kg and 300 mg/kg of AG pretreatment markedly inhibited hepatocyte damage and liver injury caused by CCl_4_ treatment (Figure 7B).

## 4. Discussion

Acute liver damage commonly occurs worldwide [3]. The pathogenesis of acute liver injury is associated with lipid accumulation, insulin resistance, inflammation, and fibrogenesis [3]. AA, a ω-6 polyunsaturated fatty acid, is an early indicator of inflammation in the development of acute hepatitis [5]. Under normal conditions, esterified AA is bound to the cell membrane’s phospholipid [24]. On the other hand, under pathological conditions, such as hepatitis, a high-fat diet, and overeating, AA is released as a free arachidonic acid from the cell membrane as a result of lipid peroxidation, thereby causing mitochondrial dysfunction and oxidative stress, including inflammatory responses and liver diseases [25]. Moreover, the liver is the primary organ that stores iron. Iron is accumulated excessively due to inflammatory syndrome or chronic liver disease [26,27,28]. Excessive iron accumulation also increases oxidative stress and lipid peroxidation, amplifying the production of reactive oxygen species (ROS) and hepatocyte damage. The release of AA is also catalyzed by excess iron [29,30]. Moreover, previous studies reported that a cotreatment of AA and iron induced cellular toxicity and mitochondrial dysfunction synergistically [12,31,32]. Here, this study also used an experimental environment, which comprises a cotreatment of AA+ iron, that causes severe oxidative stress.

AA + iron induced oxidative stress and mitochondrial damage to the cells. Normally, an over abundance of ROS is related to the dysfunction of mitochondria, mitochondrial membrane damage, and the breakdown of the redox system on the mitochondrial membrane, which is responsible for both ROS contributing and ROS scavenging at the cellular level. Excessive ROS production, which is caused by oxidative stress, is produced when inflammation and disease occur. The consequential change in redox homeostasis results in pathological conditions, including apoptosis and cellular damage to the liver [33]. ROS and mitochondrial damage were detected by FACS analysis, for which the changes also blocked the AG pretreatments.

AMPK regulates lipid homeostasis during the pathogenesis of NAFLD. Under normal conditions, the lipid metabolism is regulated by the cellular energy level, which was monitored by AMPK [34,35]. AMPK consists of a catalytic α subunit and regulatory beta and gamma units [36]. AMPK is regulated by the AMP/ATP ratio [37]. Under cellular energy starvation conditions, AMPK is phosphorylated in the threonine residue site in the AMPKα unit [38]. When the energy level is low, AMPK switches off the ATP-consuming mechanisms, such as fatty acid synthesis and protein synthesis, whereas it switches on ATP-producing mechanisms, such as fatty acid oxidation and glycolysis [39,40]. AMPK activation reflects the cellular energy level and prevents the accumulation of excessive lipids in the liver [41].

The up- and down-signaling of AMPK was reported. First, the LKB1-AMPKα signaling pathway is associated with oxidative stress. Previous studies reported that LKB1is the major upstream kinase of AMPK. LKB1 is a tumor suppressor and plays a role in the regeneration and proliferation of hepatocytes [42]. LKB1 is a cellular enzyme that activates AMPK through phosphorylation. Activated AMPK phosphorylates acetyl-coenzyme A carboxylase (ACC), a downstream signal of AMPK, activating and determining cell death or survival [22]. The LKB1-AMPKα-ACC signaling pathway can be affected by oxidative stress. Second, AMPK mediates the regulation of the YAP–Hippo pathway following cellular energy stress [43]. AMPK is linked to the Hippo–YAP pathway in cellular energy statuses. An uncontrolled Hippo–YAP pathway is observed in carcinogenesis [43]. The Hippo pathway includes Lats ½ kinases and a downstream effector, YAP, as the core components [43]. In this study, AG significantly activated LKB1-dependent AMPK signaling and the YAP pathway, which was also related to its effects against oxidative stress in the cells.

The effects of AG against CCl_4_ toxicants were demonstrated in mice. Drug-induced toxicity causes hepatic injury in mice. CCl_4_ is a direct hepatotoxin used widely in laboratories to induce acute liver disease because its biotransformation is catalyzed primarily by cytochrome P450s in hepatocytes [44,45,46]. The cytochrome P450-dependent metabolism of CCl_4_ yields trichloromethyl radical (•CCl3) and trichloromethyl peroxyl radical (•CCl3OO) as major metabolites [47], which can lead to lipid peroxidation by reacting with membrane lipids and further oxidize other cellular components, including nucleic acids and protein, resulting in hepatocyte toxicity with the release of hepatic enzymes, such as alanine aminotransferase (ALT), aspartate aminotransferase (AST), alkaline phosphatase (ALP) and bilirubin [44,48,49]. CCl_4_ treatment contributes to metabolic dysfunction including lipid peroxidation, increasing ROS production [50]. Reactive metabolites produced from CCl_4_ may cause radical stress and contribute to apoptosis, which may be responsible for hepatotoxicity and liver failure [45]. Hence, the CCl_4_ exposure model is extremely close to mimicking the cellular environment in animals from oxidative stress to carcinogenesis. The CCl_4_ exposure model was used to investigate this hypothesis to discover the novel mechanisms for therapeutic treatments against liver toxicity. In this study, CCl_4_ stimulation markedly increased plasma and histochemical parameters significantly, which was also blocked by an oral treatment of AG.

## 5. Conclusions

In conclusion, AG inhibited the cellular damage induced by AA + iron. It also inhibited the mitochondrial damage and ROS production, which was mediated by the AMPK-YAP signaling pathway in vitro. Among the components in the AG, decursin had active effects on oxidative stress and AMPK signaling. In mice, an oral treatment of AG also protected the liver, which means that AG has potential as a liver protectant against oxidative stress.

## Data Availability

The data presented in this study are available on request from the corresponding author.
